# Embracing Complexity beyond Systems Medicine: A New Approach to Chronic Immune Disorders

**DOI:** 10.3389/fimmu.2016.00587

**Published:** 2016-12-12

**Authors:** Anje A. te Velde, Tjitske Bezema, Antoine H. C. van Kampen, Aletta D. Kraneveld, Bert A. 't Hart, Henriët van Middendorp, Erik C. Hack, Joris M. van Montfrans, Clara Belzer, Lilian Jans-Beken, Raymond H. Pieters, Karen Knipping, Machteld Huber, Annemieke M. H. Boots, Johan Garssen, Tim R. Radstake, Andrea W. M. Evers, Berent J. Prakken, Irma Joosten

**Affiliations:** ^1^Tytgat Institute for Liver and Intestinal Research, Academic Medical Center, Amsterdam, Netherlands; ^2^Immunowell Foundation, Utrecht, Netherlands; ^3^Bioinformatics Laboratory, Clinical Epidemiology, Biostatistics and Bioinformatics (KEBB), Academic Medical Center, Amsterdam, Netherlands; ^4^Biosystems Data Analysis, Swammerdam Institute for Life Sciences (SILS), University of Amsterdam, Amsterdam, Netherlands; ^5^Division of Pharmacology, Utrecht Institute for Pharmaceutical Sciences, Faculty of Science, Utrecht University, Utrecht, Netherlands; ^6^Institute for Risk Assessment Sciences, Faculty of Veterinary Medicine, Utrecht University, Utrecht, Netherlands; ^7^Department of Immunobiology, Biomedical Primate Research Centre, Rijswijk, Netherlands; ^8^Department of Immunology, Erasmus University Medical Center, Rotterdam, Netherlands; ^9^Department of Neuroscience, University Medical Center, University of Groningen, Groningen, Netherlands; ^10^Institute of Psychology, Health, Medical, and Neuropsychology Unit, Faculty of Social and Behavioural Sciences, Leiden University, Leiden, Netherlands; ^11^Laboratory of Translational Immunology, University Medical Center Utrecht, Utrecht, Netherlands; ^12^Division of Pediatrics, Pediatric Immunology and Infectious Diseases, Wilhelmina Children’s Hospital, University Medical Center Utrecht, Utrecht, Netherlands; ^13^Laboratory of Microbiology, Wageningen University, Wageningen, Netherlands; ^14^Department of Psychology and Educational Sciences, Open University, Heerlen, Netherlands; ^15^Institute for Life Sciences and Chemistry, HU University of Applied Sciences Utrecht, Utrecht, Netherlands; ^16^Immunology Platform, Nutricia Research, Utrecht, Netherlands; ^17^Institute for Positive Health, Amersfoort, Netherlands; ^18^Department of Rheumatology and Clinical Immunology, University Medical Center Groningen, University of Groningen, Groningen, Netherlands; ^19^Laboratory of Translational Immunology, Department of Rheumatology and Clinical Immunology, University Medical Center Utrecht, Utrecht, Netherlands; ^20^Laboratory of Translational Immunology, Division of Pediatrics, Wilhelmina Children’s Hospital, University Medical Center Utrecht, Utrecht, Netherlands; ^21^Laboratory of Medical Immunology, Department of Laboratory Medicine, Radboud University Medical Centre, Nijmegen, Netherlands

**Keywords:** chronic immune disorders, common pathways, data integration, life style, psychosocial factors

## Abstract

In order to combat chronic immune disorders (CIDs), it is an absolute necessity to understand the bigger picture, one that goes beyond insights at a one-disease, molecular, cellular, and static level. To unravel this bigger picture we advocate an integral, cross-disciplinary approach capable of embracing the complexity of the field. This paper discusses the current knowledge on common pathways in CIDs including general psychosocial and lifestyle factors associated with immune functioning. We demonstrate the lack of more in-depth psychosocial and lifestyle factors in current research cohorts and most importantly the need for an all-encompassing analysis of these factors. The second part of the paper discusses the challenges of understanding immune system dynamics and effectively integrating all key perspectives on immune functioning, including the patient’s perspective itself. This paper suggests the use of techniques from complex systems science in describing and simulating healthy or deviating behavior of the immune system in its biopsychosocial surroundings. The patient’s perspective data are suggested to be generated by using specific narrative techniques. We conclude that to gain more insight into the behavior of the whole system and to acquire new ways of combatting CIDs, we need to construct and apply new techniques in the field of computational and complexity science, to an even wider variety of dynamic data than used in today’s systems medicine.

## Introduction

Chronic immune disorders (CIDs), comprising chronic immune-mediated inflammatory conditions, such as autoimmune diseases, allergies, immune deficiencies, and some psychiatric disorders (such as depression) are a large and growing health problem. Approximately 1 in 10 individuals living in Europe and North America are affected, and consequently, CIDs represent a significant cause of chronic morbidity and disability (1, 2), strongly impacting the quality of life.

This translates into a substantial (socio)economic challenge to rapidly improve prediction, prevention, diagnosis, and treatment of these diseases in order to significantly reduce health-care costs^1^ (3–5).

More than 50 years of immunological research has brought us detailed insights into immune pathways at both a molecular and cellular level but still leaves fundamental questions unanswered: which (common) factors contribute to the onset of CIDs and which mechanisms keep the immune system in homeostasis or can drive the system into disease states?

In order to combat CIDs, we need to understand the bigger picture that goes beyond insights at a one-disease, molecular, cellular, and static level. To unravel this bigger picture we should use an integral, cross-disciplinary approach (6, 7). Although the idea of such an approach is not entirely new, most challenges to transform it into truly integral scientific pan-disease projects are still to be met. It is crucial to embrace the complexity of the CID field and boldly start applying new techniques from the field of complex systems science to effectively combine different scientific perspectives, including the human (patient) perspective.

## The Bigger Picture

**Figure 1:**
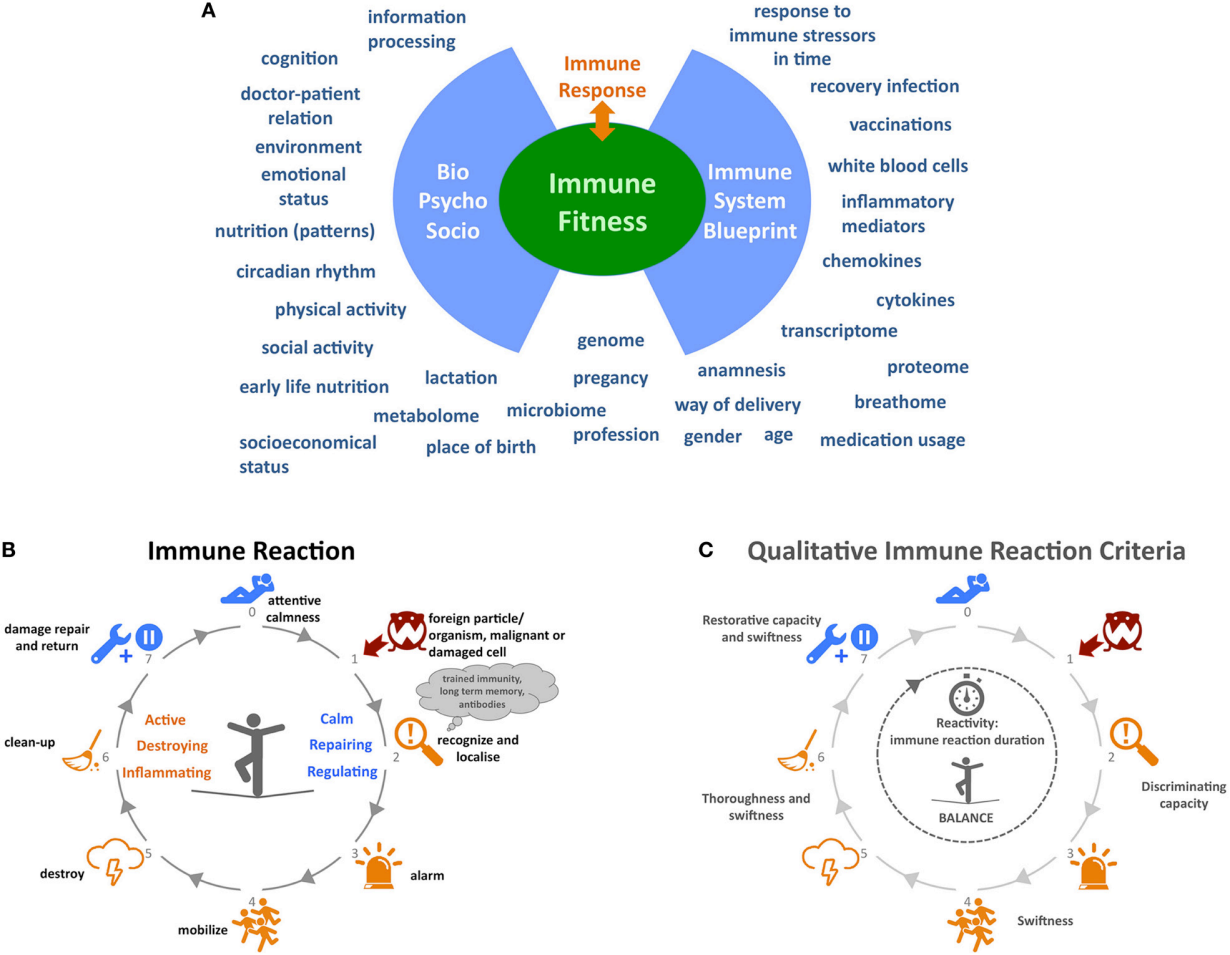
**Immune functioning: embracing complexity**. **(A)** A wide variety of determinants influencing immune function. **(B)** Process overview of the immune system behavior. **(C)** Qualitative criteria for the behavior of the immune system. At step 2 “recognize and localize” the system must be able to discriminate between true threats and harmless organisms or cells. The discriminating capacity of the system might be low in disorders like allergies, some autoimmune diseases, and immunodeficiencies. Between steps 3 and 5, the swiftness or speed of alarming, mobilizing, and destroying is key for the immune system’s effectiveness in combatting pathogens before they start growing or mutating. The same applies to the thoroughness of destroying and cleaning up pathogens in steps 5 and 6 so that no or very little strain on the body remains after the infection. The capacity to restore any damage after the infection at step 7 and the speed at which the system returns to an attemptive calmness is an often forgotten aspect of the immune system’s capabilities. In autoimmune diseases, this might be disrupted. In conclusion, the whole duration of the immune reaction to a certain pathogen can also be an indicator for (un)healthy behavior. Icons in panels **(B,C)** made by Katarina Stefanikova (lightning cloud) and Freepik (other icons) are from www.flaticon.com.

In order to gain more insight into the driving mechanisms underlying CIDs we need to broaden our current reductionistic focus on molecular, cellular, and organ level of a single CID to a holistic strategy that considers multiple CIDs and incorporates the microbiome, psychological, social, and lifestyle determinants. Although several projects and consortia exist worldwide that incorporate some of the aforementioned immune parameters and general psychosocial aspects, a number of key factors are lacking. An all-encompassing analysis is needed. Here, we will provide a more detailed insight into factors that in our opinion could contribute to this bigger picture (Figure 1A).

## Common Pathways in CIDs

The existence of common pathogenic pathways in different types of CIDs indicates that in addition to focusing on single CID research we should make an effort to understand the commonalities of CIDs. In recent years, a large number of genome-wide association studies (GWAS) for CIDs have been performed to identify single nucleotide polymorphisms (SNPs) associated with disease phenotypes. Several hundreds of disease-associated SNPs have been identified including a large number of variants that are shared between CIDs (8–10). For the interpretation of the GWAS findings, the first steps are taken to understand the function of individual genes, their interactions, and to get insights into how these genetic variants are associated with the underlying immune disease (11–16). Follow-up studies should also incorporate epigenetics, transcriptomics, proteomics, and genome engineering to understand the functional relevance of SNPs (17, 18). Patient stratification based on cell-specific transcriptomic data was recently reported (19, 20). In addition, comorbidity of immune disorders, for example, in patients with inflammatory bowel diseases, psoriasis, or rheumatoid arthritis (21, 22) demonstrates that common mechanisms probably underlie immune disorders (23). Recent work in the field of systems medicine (24, 25) shows commonalities at the molecular and cellular level between subgroups of patients diagnosed with different CIDs. Also, indications that most of the CIDs share the same effector mechanisms and pathways of inflammation can be exemplified by the observation that specific therapeutic approaches in one specific CID can often be used in another CID as well. Examples are the use of anti-TNFα treatment in rheumatoid arthritis, psoriasis, and IBD (26–28) and targeting the IL-12/IL-23 pathway in various other CIDs (29). We can conclude that, despite the presentation of different clinical symptoms and involvement of different organs, distinct CIDs share common immune mechanisms.

### General Psychosocial and Lifestyle Factors Associated with Immune Functioning

Next to genetic or physiological processes related to immune functioning, other general factors might impact immune functioning in both healthy individuals and patients with CIDs. It is important to look into the evidence regarding the role of psychosocial and lifestyle factors on different elements of immune system functioning, as an insight into these factors may provide leads to improved immune functioning in immunocompromised populations by means of non-invasive, non-medical strategies.

### Psychosocial Factors

There is convincing evidence supporting a role of psychosocial factors in influencing immune processes and inflammatory function in both healthy and immunocompromised conditions. For example, since the seminal paper by Cohen and colleagues (30), consistent evidence has been reported for the link between both acute and chronic psychological stress and the suppression of immune system functioning (e.g., percentage of T suppressor/cytotoxic cells, levels of pro-inflammatory cytokines, and antibody response to immunization) (31–33). Next to the role of stress exposure, cognitive behavioral ways of dealing with stress, such as more passive-avoidant coping, and social resources, for instance, lack of social support, influence longer-term immune-related outcomes, such as disease progression, as shown for example in natural course studies of acute infections or patients with chronic inflammatory diseases (31). Although more research is needed into the mechanisms that underlie the association between psychological factors and immune system functioning, the available evidence suggests that corresponding psychosocial factors impact generic inflammatory parameters of immune function and as such are potentially useful factors for intervention in order to improve immune functioning.

In line with the evidence for psychosocial factors as potentially impacting on immune function, psychosocial interventions, particularly stress-management interventions and cognitive behavioral therapies, have been shown to affect indicators of immune function, such as lymphocyte counts, pro-inflammatory cytokine levels, or inflammatory activity (32–34). Evidence for the beneficial effects of interventions at this level is somewhat less consistent than for stress research, particularly due to the lower number of studies systematically measuring the same immune-related outcome parameters. Corresponding findings across diseases for both psychosocial factors, such as stress, and psychosocial interventions, such as stress-management interventions, suggest the involvement of common psychosocial mechanisms both in a fit and disturbed immune function, independent of the disease-specific pathogenic mechanisms.

At a molecular and cellular level several known neuro–immune interactions also support these findings. For instance, neurons produce cytokines and express “immunological receptors” like toll-like, immunoglobulin, cytokine, and chemokine receptors. *Vice versa*, immune cells produce neuropeptides and neurotransmitters and express their receptors (35, 36). The bidirectional loop between immune and nervous system was also highlighted by the fact that CID patients have an elevated risk for depression (37–40).

In view of the common psychological mechanisms that seem to play a role for both a fit and disturbed immune function, independent of the disease-specific pathways, integrated research strategies across conditions are needed. However, knowledge about possible common processes is not yet systematically used and integrated in the disease-oriented research traditions. Consequently, different lines of research usually independently exist, and exchange of knowledge across diseases or for both diseases and well-functioning immune function is very limited. Integration of knowledge from different disciplines enables an integrated theoretical and clinical approach that takes into account both the role of disease-specific and generic psychosocial and biological factors as well as their possible interactions in stimulating and disturbing immune function for both healthy subjects and patients, with the ultimate goal to better understand immune function across health and disease.

### Lifestyle Factors

We are what we eat and where or how we live (41). The environment shapes our immune system. It is now well-known that lifestyle (e.g., diet, drinking) has an enormous influence on our immune system directly or indirectly through the microbiome (42–46). Also, the duration of exposure to lifestyle factors (our age) is an important factor, as are many other mostly under-investigated factors such as sleep rhythm. To demonstrate just the tip of the iceberg in lifestyle factors potentially impacting on immune function, two factors are discussed here, intestinal microbiome and physical activity.

The intestinal microbiota contributes to human health through its ability to release energy and nutrients from food as well as regulating host immune and metabolic functions (47). The microbiota lives in symbiosis with the host. In the adult human, the two main phyla are Firmicutes, Gram-positive bacteria, and Bacteroidetes, Gram-negative bacteria (48). The development of the intestinal microbiota blueprint occurs in the first 1,000 days of life and is a dynamic process influenced by mode of delivery, antibiotic use, and early-life nutrition (49). Many experimental models show that the gut microbiota is vital for normal immune development and regulation (50). Pioneer bacteria colonizing the infant intestinal tract and gradual diversification toward a stable gut ecosystem play a crucial role in establishing stable host–microbe interactions and an optimal symbiosis between them. A crucial step in healthy postnatal microbiota development is the developing mucosal immune system that distinguishes between beneficial and pathogenic (microbial) entities. As such, an altered microbiota composition in early or later life can be linked to a number of CIDs (51). In addition, there is a parallel and interacting microbiota and immune system development throughout the life span with critical periods such as early life, but also adolescence (52–54) and old age (55–58), the latter possibly more involved in late-onset CIDs. Although multifactorial aspects are involved in CIDs, changes in microbiota and activity are implicated in a broad range of these chronic conditions (43). The altered intestinal colonization patterns in CIDs are associated with reduced microbial diversity as a common denominator. All of this is regarded as dysbiosis: “an imbalanced microbiota composition and/or activity, which disrupts the host–microbiota homeostasis” (59, 60), resulting in an overactive immune response associated with CIDs.

An additional environmental factor with possible pronounced effects on the function of the immune system is physical activity (61). It is, for instance, often reported that walking a marathon is not healthy, which has been confirmed in studies in recreational marathon runners showing a number of adverse changes in diverse immune system components that may leave the individual more susceptible to infectious diseases for a short time period (62). In line with this, several immune parameters have been shown to be suppressed when people train intensively for a prolonged period of time, including decrease of neutrophil function, immunoglobulin concentration, and natural killer cell counts, being associated with increased infection susceptibility (61, 63–65). On the other hand, epidemiological data show that regular moderate physical activity contributes to the prevention of disease and promotion of health (63, 64). Recently, in a systematic review based on studies examining PBMC gene expression, it has been demonstrated that prolonged and regular physical activity promotes inflammation dampening effects, possibly resulting in a reduced risk for the development or exacerbation of CIDs (66).

## Challenges: Embracing Complexity

### Integrating and Extending Data and Including Immune System Dynamics

Considering the immune system, there is still a giant leap between the current limited sets of parameters determined in the various consortia (67–70) and the actual usage of the data for integration, as depicted in Table 1, there are many parameters of the immune system that can be measured, but most initiatives limit measurements to a subset of parameters based on genetics or immune phenotyping.

**Table 1:** **Scope of Consortia**.

Parameters	Omics				Immune			Psychosocial				
	Genome/exome sequencing/SMPs	Epigenetics/transcriptome Variation	Proteomics/metabolomics	Microbiomics	Vaccination response	Immune (cell) phenotyping	Cytokine/chemokine variation	Socioeconomic data	Quality of life	Stress perception and coping	Personality characterization	Life style (f.i. diet, sleep exercise)
Human Microbiome Project http://hmpdacc.org												
The Immunological Genome Project (mouse) https://www.immgen.org												
Immvar http://www.immvar.org/ImmVar.swf												
Personal Genome Project http://www.personalgenomes.org												
Genotype Tissue Expression Consortium http://www.gtexportal.org												
Stanford Medicine Centers http://med.stanford.edu												
100k Wellness Project https://www.systemsbiology.org												
Milieu Intérieur Consortium http://www.milieuinterieur.fr												
Human Functional Genomics Project http://humanfunctionalgenomics.org												

*Data derived from the indicated websites*.

The main paradox in most current approaches is that measuring static immune function parameters does not comply with the fact that the immune system is a reactive as well as a dynamic system. Checking a static situation by a single measurement of serum proteins at a certain point in time does not reflect the response capacities of an individual’s immune system. An already well-explored option is to perform longitudinal *ex vivo* experiments with (isolated) blood or tissue-derived cells of the person in question and expose these cells to a number of different relevant immune stimuli, such as bacterial and viral compounds or immune proteins (cytokines or immunoglobulins) (71). An obvious, albeit inevitable, limitation of this approach is that the cells are removed from their physiological context and placed in an artificial culture system. Nevertheless, these experiments can show us the dimensions and variability of the response to distinct immunological stimuli. From these data, network representations of the immune system that reflect the dynamic feedback-regulated interactions involving many different components can be constructed (72). In addition, we will need to include as much information as possible about (epi)genetic, transcriptomic, proteomic, environmental, and psychosociological factors. When including the last mentioned factors into the equation, human research is inevitable and calls for new techniques from the field of big data science to be able to identify essential interactions between the immune system and its internal and external surrounding systems. Furthermore, new techniques are needed to describe and simulate the behavior of the immune system as a complex adaptive system. For this we suggest to apply complex adaptive systems science, for example, those that are successfully applied in studying and modeling of ecosystems (73).

### Measures for Healthy Systems Behavior

In its interaction with other physiological systems in the body and its versatile reaction to external factors, the immune system of a person might show typical behaviors that could indicate susceptibility to certain CIDs. The systematic investigation of this system behavior requires measurement methods that include the system’s dynamics.

In 2008, Mark Davis, immunologist at Stanford University, asked the question “How is my immune system?” and elaborated on the fact that we are unable to give an answer, because we lack metrics of immunological health in humans (74). To develop such metrics, we need to start developing meaningful metrics for the behavior, adaptiveness, and responsiveness of the system as a whole. These metrics could be a measure for the “fitness” of the immune system. Currently, our knowledge does not allow us to determine the immune fitness of an individual and, consequently, it is impossible to determine how (much) an individual patient’s immune function deviates from a fit immune system. Such a view would also help us to define and understand different degrees of immune fitness. The big immunological picture will enable us to treat patients much more efficiently and effectively and successfully revert the “sick” immune system to a fit state. It will also help healthy individuals to stay fit. The depicted immune reaction process in Figure 1B is a simplified attempt to describe the behavior, adaptiveness, and responsiveness of the system as a whole, in order to demonstrate that a different approach can lead to helpful new ideas. In Figure 1C, a selection of qualitative criteria are shown that are derived from this approach and that could be developed into quantitative and normative criteria for the behavior of the immune system.

### The Need to Integrate Experience-Based Knowledge

We are in great need for innovative multidisciplinary scientific research that enriches immunological knowledge with experience-based knowledge of a large group of CID patients, which incorporates a broad view on health, including psychosocial and lifestyle aspects (75).

A way to integrate experience-based knowledge into scientific research directly from the source (the patients themselves) is to translate patient narratives into hypotheses and include data from the narratives into the all-encompassing analyses. By designing a specific mix of qualitative, story-provoking questions and quantitative questions that have been designed to add meaning to certain aspects of their narratives, patients are invited to tell about their experiences with their CID(s) from their own perspective (76). For instance, patients could tell about life events that might be associated with their CID(s) or lifestyle factors that, to their experience, influence their disease activity. When a sufficient number of narratives will be collected, an evaluation and analysis of the narratives can point out certain patterns that emerge. If both the design of the questions and the evaluation of the narratives are done by a group that includes not only scientists and specialists but also patients, this bottom-up “participatory narrative inquiry” method is a powerful method for gaining unexpected insights and leads in the complex domain of CIDs.^2^

For the analysis of (extended) immune system (sub)phenotypes, we need to collect multiple data from well-defined patients and healthy volunteer cohorts, using a multidisciplinary and longitudinal approach. Collaboration between immunologists, computational biologists, patients, and their family suffering from CID(s), computer scientists, health-care professionals, physicians, psychologists, sociologists, physiologists, physicists, pharmacologists, pharmacists, microbiologists, dieticians, and others is essential to find creative ideas and solutions to complex problems [see also Ref. (77) on collaborations]. Current genotype–phenotype databases have their limitations because they are generally disease-focused, not publicly available, and contain incomplete data and annotation (78). We should collect as many individual data as we can, without overlooking things because of prior exclusion of supposedly irrelevant information or only include those hypothesized to be directly involved, to be able to find new subtypes as well as common pathways of CIDs, and additionally possible prevention strategies and new ways of treatment (79). In this way, we will refrain from the reductionist view that is based on incomplete knowledge of the biological mechanisms of gene functions in isolated cells and tissues or in inbred mice. From the analysis of these data, new hypotheses can be generated (80). To ensure transparency, reproducibility, and reusability, the data management should be guided by the recently published FAIR (Findability, Accessibility, Interoperability, and Reusability) data principles (81).

Both top-down and bottom-up approaches (82–84) can be used to investigate the commonalities between different CIDs. Using top-down approaches and data from public resources, one may attempt to reconstruct biological networks that are common in multiple CIDs. Alternatively, bottom-up approaches can be used to develop and integrate smaller but more detailed mathematical models to understand properties of the immune system. The integration of psychosocial factors is only possible when data are available from studies in which these factors were actually assessed, which generally will not be the case. Therefore, we believe that additional large-scale studies are required to investigate the effects of such factors on biological networks. Bottom-up approaches may also be used to study perturbation of disease pathways involved in comorbidity. This requires knowledge of such pathways. (Hypothesized) Psychosocial effects might be included in such mathematical models. To the best of our knowledge, top-down and bottom-up approaches have not yet been used to study commonalities in CIDs. Using these approaches, the comparison of patients with CIDs and healthy individuals may lead to a better definition of immune fitness. With this approach, we might also be able to learn to understand the physiology of unexplained complaints such as discomfort and fatigue.

Once we were able to determine the boundaries and dynamics of an adequate immune reaction, it is possible to identify aberrances in patients with CIDs. In this way, we might find an answer to the question whether derailing immunity is a consequence of a high (or low) responsive state in groups of patients determined by a certain genetic and environmental make-up. By doing so, we can develop comprehensive risk models that integrate a certain immune status with extensive omics and a broad environmental risk profiles that includes cognitive, emotional, dietary, and social risk factors. This will result in tools such as biomarkers and predictive algorithms that can support prevention and clinical management.

## Conclusion

In this position document, we propose the concept that elucidating treatment and prevention of CIDs can be much accelerated when we start connecting diseases to underlying processes and place disease mechanisms at a molecular and cellular level in a much broader perspective. In order to understand which (common) factors contribute to the onset or exacerbation of CIDs and which mechanisms keep the immune system in homeostasis or drive the system into disease states, components such as life style, emotions, cognitions, and behaviors must also be incorporated. Current computational and complexity science will allow us to generate insight into the complex behavior of the whole system if we dare to start developing new techniques from this field and apply it to unravel the big picture.

## Author Contributions

All the authors contributed to the conception and design of the work and interpretation of the data; AV, TB, AvK, AK, AE, HM, CB, and IJ did the acquisition and analysis of the data and drafted the work; and BH, EH, JM, LJ-B, RP, KK, MH, AB, JG, TR, and BP revised it critically for important intellectual content. All the authors approved the final version of the manuscript and agreed to be accountable for all aspects of the work.

## Conflict of Interest Statement

The authors declare that the research was conducted in the absence of any commercial or financial relationships that could be construed as a potential conflict of interest.

## Footnotes

^1^http://www.who.int/ncds/introduction/en/.

^2^http://www.workingwithstories.org/WorkingWithStoriesThirdEdition_Web.pdf.
